# Supplementary material for: The Experiences and Perspectives of Persons with Prostate Cancer and Their Partners: A Qualitative Evidence Synthesis Using Meta-Ethnography
Source: Healthcare (Basel). 2024 Jul 27;12(15):1490. doi: 10.3390/healthcare12151490 (PMC11311449; doi:10.3390/healthcare12151490)
Supplement: Supplementary file 1 [file healthcare-12-01490-s001.zip › healthcare-3003740-supplementary.pdf]

Supplementary File: eMERGe reporting guidelines.

No	Criteria Headings	Reporting Criteria	Report Location
Phrase 1: Selecting the meta-ethnography and getting started			
Introduction			
1	Rational for context for meta-ethnography	Describe the gap in research or knowledge to be filled by the meta-ethnography, and the wider context of the meta-ethnography	Page 1
2	Aims of the meta-ethnography	Describe the meta-ethnography aim(s)	Page 1
3	Focus of the meta-ethnography	Describe the meta-ethnography review question(s) (or objectives)	Page 1and 2
4	Rationale for using meta-ethnography	Explain why meta-ethnography was considered the most appropriate qualitative synthesis methodology	Page 2
Phrase 2- Deciding what is relevant			
Methods			
5	Search strategy	Describe the rationale for the literature search strategy	Page 2
6	Search Processes	Describe how the literature searching was carried out and by whom	Page 3
7	Selecting primary studies	Describe the process of study screening and selection, and who was involved	Page 3
Findings			
8	Outcome of the study	Describe the results of study searches and screening	Page 4
Phrase 3- Reading Included studies			
Methods			

9	Reading and data extraction approach	Describe the reading and data extraction method and processes	Page 3
Findings			
10	Presenting characteristics of included studies	Describe the characteristics of the included studies	Page 4
Phrase 4- Determining how the studies are related			
Methods			
11	Process for determining how studies are related	Describe the methods and processes for determining how the included studies are related:<br>Which aspects of studies were compared<br>AND<br>How the studies were compared	Page 4
Findings			
12	Outcome of relating studies	Describe how the studies relate to each other	Page 4
Phrase 5-Translating studies into one another			
13	Process of translating studies	Describe the methods of translation:<br>-Describe steps taken to preserve the context and meaning of the relationships between concepts within and across studies- Describe how the reciprocal and refutational translations were conducted- Describe how potential alternative interpretations or explanations were considered in the translations	Page 5
Findings			

14	Outcome of translation	Describe the interpretive findings of the translation.	Page 5 and 6
Phrase 6- Synthesizing translations			
Methods			
15	Synthesis Process	Describe the methods used to develop overarching concepts ("synthesised translations")<br><br>Describe how potential alternative interpretations or explanations were considered in the synthesis	Page 5 and 6
Findings			
16	Outcome of synthesis process	Describe the new theory, conceptual framework, model, configuration or interpretation of data developed from the synthesis	Page 8
Phrase 7-Expressing the synthesis			
Discussion			
17	Summary of findings	Summarize the main interpretive findings of the translation and synthesis and compare them to existing literature	Page 9
18	Strengths, limitations, and reflexivity	Reflect on and describe the strengths and limitations of the synthesis:<br><br>- Methodological aspects—for example, describe how the synthesis findings were influenced by the nature of the included studies and how the meta-ethnography was conducted.	Page 10

		- Reflexivity—for example, the impact of the research team on the synthesis findings	
19	Recommendations and conclusion	Describe the implications of the synthesis	Page 10
